# Was Dinosaurian Physiology Inherited by Birds? Reconciling Slow Growth in *Archaeopteryx*

**DOI:** 10.1371/journal.pone.0007390

**Published:** 2009-10-09

**Authors:** Gregory M. Erickson, Oliver W. M. Rauhut, Zhonghe Zhou, Alan H. Turner, Brian D. Inouye, Dongyu Hu, Mark A. Norell

**Affiliations:** 1 Department of Biological Science, Florida State University, Tallahassee, Florida, United States of America; 2 Bayerische Staatssammlung für Paläontologie und Geologie and Department of Earth and Environmental Sciences, LMU Munich, München, Germany; 3 Key Laboratory of Evolutionary Systematics of Vertebrates, Institute of Vertebrate Paleontology & Paleoanthropology, Chinese Academy of Science, Beijing, China; 4 Department of Anatomical Sciences, Stony Brook University, Stony Brook, New York, United States of America; 5 Paleontological Institute, Shenyang Normal University, Shenyang, China; 6 Division of Paleontology, American Museum of Natural History, New York, New York, United States of America; American Museum of Natural History, United States of America

## Abstract

**Background:**

*Archaeopteryx* is the oldest and most primitive known bird (Avialae). It is believed that the growth and energetic physiology of basalmost birds such as *Archaeopteryx* were inherited in their entirety from non-avialan dinosaurs. This hypothesis predicts that the long bones in these birds formed using rapidly growing, well-vascularized woven tissue typical of non-avialan dinosaurs.

**Methodology/Principal Findings:**

We report that *Archaeopteryx* long bones are composed of nearly avascular parallel-fibered bone. This is among the slowest growing osseous tissues and is common in ectothermic reptiles. These findings dispute the hypothesis that non-avialan dinosaur growth and physiology were inherited in totality by the first birds. Examining these findings in a phylogenetic context required intensive sampling of outgroup dinosaurs and basalmost birds. Our results demonstrate the presence of a scale-dependent maniraptoran histological continuum that *Archaeopteryx* and other basalmost birds follow. Growth analysis for *Archaeopteryx* suggests that these animals showed exponential growth rates like non-avialan dinosaurs, three times slower than living precocial birds, but still within the lowermost range for all endothermic vertebrates.

**Conclusions/Significance:**

The unexpected histology of *Archaeopteryx* and other basalmost birds is actually consistent with retention of the phylogenetically earlier paravian dinosaur condition when size is considered. The first birds were simply feathered dinosaurs with respect to growth and energetic physiology. The evolution of the novel pattern in modern forms occurred later in the group's history.

## Introduction

**Figure 1:**
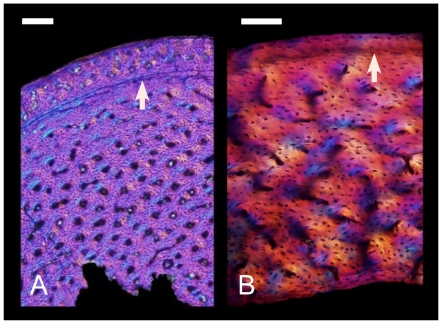
Long bone histology of a non-avialan dinosaur and a Mesozoic bird viewed with polarized microscopy. (A) The femoral microstructure of the small alvarezsaurrid, *Shuvuuia deserti* (IGM 100–99) is compared to the tibial histology (B) of the Early Cretaceous avialan, *Confuciusornis sanctus* (IVPP V11521). Both show well-vascularized bone owing to the presence of numerous primary vascular canals (large black structures), and woven fibered matrix characterized by oblong, randomly oriented osteocyte lacunae (numerous small black structures). Arrows point to growth lines in the form of a line of arrested growth (left) and an annulus (right). Scale bars  = 0.5 mm (A) and 0.1 mm (B).

The genesis of birds was a key event in vertebrate history. The descendants of these animals have dominated aerial niches from the Late Mesozoic until today, where they are the most speciose amniote clade (∼10,000 species) [1], [2]. Skeletal, feather, and behavioral evidence conclusively place birds within the evolutionary radiation of theropod dinosaurs, rightly making birds the only living dinosaurs [2]–[4]. Growth patterns, revealed through long bone osteohistology can be traced phylogenetically through this transition and used as a proxy for inferring physiological changes. Non-avialan dinosaur bones show a characteristic well-vascularized, rapidly formed woven-fibered matrix that is often interrupted by growth lines [5]–[7] (Figure 1). Similar broad-scale histological attributes have been reported in Mesozoic bird lineages close to the base of the avialan tree [8]–[11] (Figure 1). This has contributed to the paradigm that dinosaurian physiology is ancestral for Avialae as a whole. (Although, whether that condition was comparable to living birds is a subject of debate [6], [12]–[15].)

**Figure 2:**
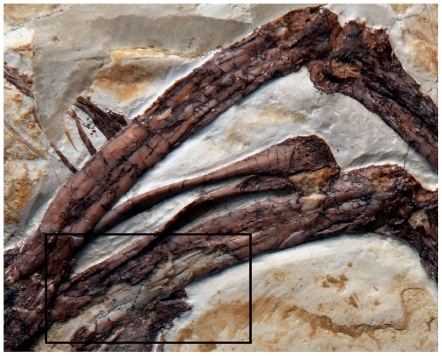
Skeletal elements from the largest known *Archaeopteryx* specimen showing cortical delamination. Femora, tibiae, fibulae, pubes, and gastralia from the Solnhofen specimen (BMMS 500), are shown. The brown cortical layer of the left femur (inset box) is superior to the light gray underlying bone layer that is exposed where the former flaked-off. This is consistent with a growth line interface. These thin, hypermineralized osseous layers partition the more fibrous zonal tissues and act as planes of weakness where exfoliation can occur.

**Figure 3:**
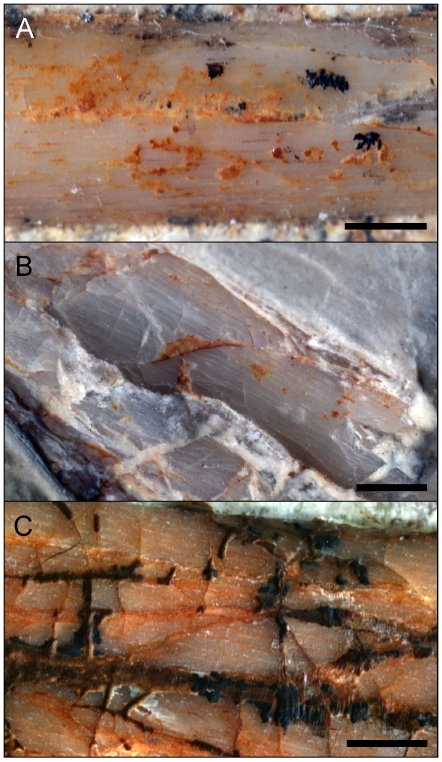
Fibrous surface texture and longitudinal vascular canals visible within the cortices of *Archaeopteryx* long bones. (A) Tibial diaphysis from the smallest known *Archaeopteryx*, the Eichstät specimen (JM 2257) showing sparse longitudinal vascularization in the form of parallel striae. Comparable immature texture and patterning is found in the major long bones throughout the *Archaeopteryx* growth series. For example, it is seen in the tibiae of the moderately larger Munich specimen (BSPG 1999 I 50) shown in (B), and in the largest known individual (The Solnhofen specimen, BMMS 500) shown in (C). Scale bars  = 1.5 mm.

We used dissecting microscopy to directly examine the long bone periosteal and fracture surfaces spanning the entire size range of *Archaeopteryx* (Eichstätt, Munich, Ottmann & Steil, London, and Solnhofen specimens; Table 1), the oldest known bird [16]. (Note: we follow Chiappe's (2) interpretation that all specimens are referable to a single species, *Archaeopteryx lithographica*; see Materials and Methods.) In the two largest individuals delamination of cortical bone consistent with growth line interfaces were found, as anticipated (Figure 2). However, all specimens unexpectedly showed exceptionally sparse longitudinal vascularization visible on the periostal surfaces and within the semi-transparent bones (Figure 3). Furthermore, transversely running fracture faces showed a circumferential fabric characteristic of the dense lamellar or parallel-fibered bone-types of living non-dinosaurian reptiles [17].

**Table 1:** Specimen identifications and sizes.

Taxon	Specimen Number(s)	Max. Femoral Length (mm)
*Citipati osmolskae*	IGM 100–1004*	405
*Conchoraptor gracilis*	IGM 97–212*	250
*Caudipteryx zoui*	IVPP 11819*	149
*Troodon formosus*	MOR 748*	483
*Byronosaurus jaffei*	IGM 100–984*	150
Troodontidae (undescribed)	IGM 100/1129*	84
Troodontidae (undescribed)	IGM 100–1323*	80
*Utahraptor ostrommaysi*	BYUVP 15465*	600
*Velociraptor mongoliensis*	IGM 100–982*	184
*Mahakala omnogova*	IGM 100–1033*	76.2
*Sapeornis chaoyangensis*	LPM B00166*	80
*Jeholornis prima*	IVPP 13274*, IVPP 13353*	75
*Archaeopteryx lithographica*	JM 2257, BSPG 1999 I 50*	65.7
	BMMS 500, BMNH 37001	
	Ottmann & Steil—uncataloged	
	TSMHN 6928/6929**	
	WDC-SG-100**	
	HMN 1880/1881**	
	Maxberg—uncataloged**	
	8^th^ Exemplar—uncataloged**	

*Histologically sampled. **Examined using research photographs from colleagues and/or through high-resolution research casts. Institution designations: BMMS  =  Bürgermeister-Müller Museum, Solnhofen; BMNH  =  Museum of Natural History, London; BSPG  =  Bayerische Staatssammlung für Paläontogie und Geologie, Munich; BYUVP  =  Brigham Young University Earth Science Museum, Provo; HMN  =  Museum für Naturkunde, Humboldt-Universität, Berlin; IGM  =  Institute of Geology, Mongolian Academy of Sciences, Ulaanbaatar; IVPP  =  Institute of Vertebrate Paleontology and Paleoanthropology, Beijing; JM  =  Jura Museum, Eichstät; LPM  =  Shenyang Normal University, Shenyang; MOR  =  Museum of the Rockies, Montana State University, Bozeman; TSMHN  =  Teyler-Museum Haarlem; WDC  =  Wyoming Dinosaur Center, Thermopolis.

These observations conflict with the hypothesis that dinosaurian-type growth physiology is primitive for the first birds. Alternative explanations are: 1) *Archaeopteryx* grew aberrantly, 2) basalmost avialan growth was substantially decelerated, perhaps in response to the reallocation of resources from growth to powered flight, or 3) that current phylogenetic resolution of histological character states are insufficient to correctly infer the basalmost avialan condition.

**Figure 4:**
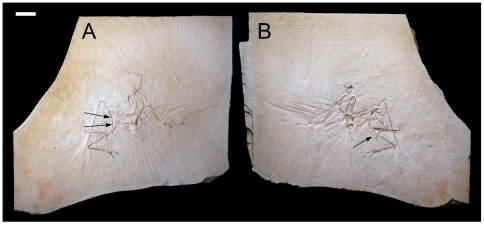
Slab and counterslab of the Munich *Archaeopteryx* (BSPG 1999 I 50). Cortical samples were extracted from the fracture faces of broken elements. (A) Main slab with arrows showing where femoral samples were extracted. (B) Counterslab showing where the fibula was sampled. Scale bar  = 5 cm.

To test these hypotheses we used a comparative phylogenetic approach. We were given the opportunity to study the long bone histology of a juvenile specimen of *Archaeopteryx* (The Munich Specimen, BSPG 1999 I 50; Figure 4) from which we describe the taxon's peculiar osseous microstructure. We also sampled the successive crownward lineages, *Jeholornis* (another very primitive long-tailed avialan; [18]), and *Sapeornis* (the primitive short-tailed avialan that retains three fingers and teeth; [19]), to provide broader representation of avialan histology in truly basal lineages (Table 1). (Initial studies utilized the histology of *Rahonavis*, that was once thought to occupy a similar position [5], [20] but is now considered a non-avialan dinosaur [21], [22]; and the birds *Confuciusornis*, Enantiornithes, and *Patagopteryx* [5], [9], [20], [23] that are now placed well crownward from the basal avialan node [24].)

Interpreting our basal avialan findings in a phylogenetic context spanning the avialan transition also necessitated broader histological sampling of the proximate outgroup maniraptoran clades, Oviraptorosauria and Deinonychosauria ([22], [24]; Figure S1). Initial examinations across the avialan transition had either no samples ascribed to these clades, or just one representative – *Troodon* [7], [23] or *Unenlagia* [5]. The large size of these deinonychosaurs (∼50–100 kg; [22]) is a potential problem for predicting the basal condition for Avialae as scaling has a strong influence on histological pattern [17], [25]–[29]. Dinosaurs are no exception [7], [27], [28] and it has recently been shown that basal non-avialan deinonychosaurs were actually miniaturized (∼1 kg) and this size is primitive for Paraves (Deinonychosauria + Avialae) [22]. These data demonstrate the need for broader sampling (both phylogenetically and inclusive of scale) among outgroup clades.

Finally, because tissue formation rates can reveal developmental rates [9], [30] we used the growth series for *Archaeopteryx* to independently test the hypothesis that non-avialan dinosaurian growth rates were ancestral for birds. The results were compared to expectations for same-sized non-avialan dinosaurs [31] and extant precocial birds [32].

## Results

**Figure 5:**
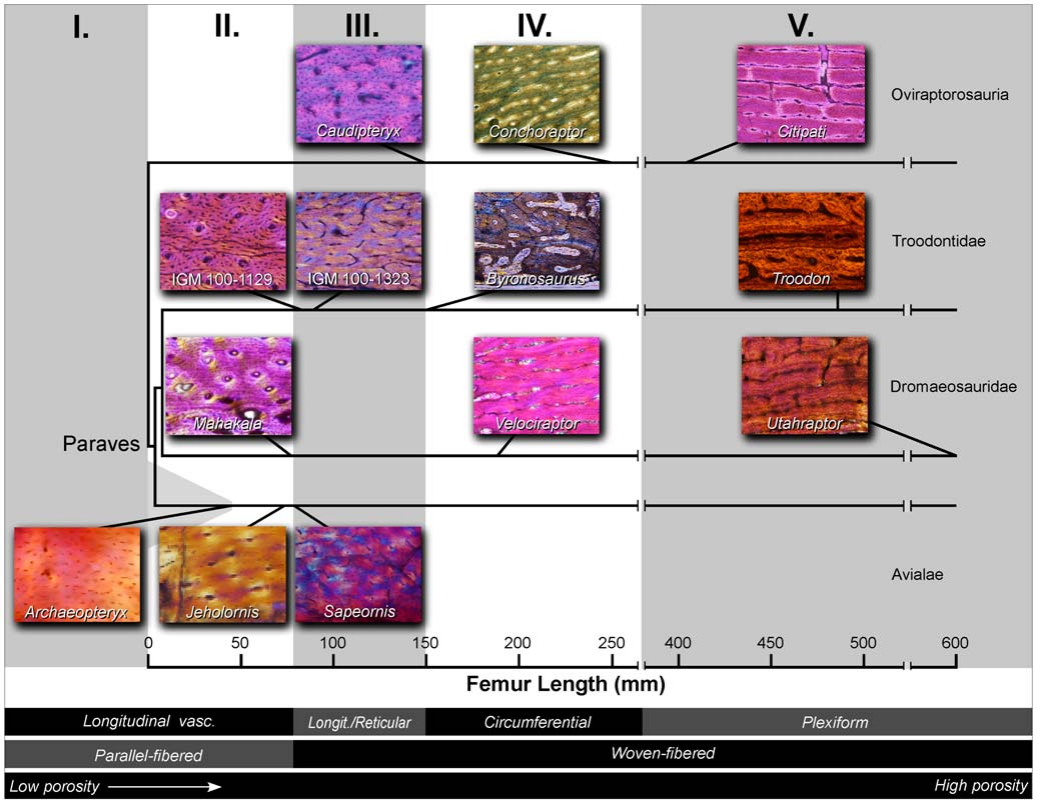
Cladogram for the Maniraptora showing character mapping of primary femoral histological types with respect to scale. The specimens are viewed with polarized microscopy. Each is oriented with the periosteal surface towards the top of the figure. Five histological character suites (I.–V.) are present. The attributes composing each suite appear below the figure and are denoted by (black and/or gray horizontal bars). For example: Maniraptoran Type I shows longitudinal vascularizaton, parallel-fibered matrix, and low porosity. Lines stemming from the bottom the histological images trace to the representative histological suites for each taxon. The character suites show intracladal scale dependence with respect to femoral length. The basal avialans conform to expectations of same-sized non-avialan maniraptoran outgroups. Phylogenetic hypothesis based on ref. 22.

**Figure 6:**
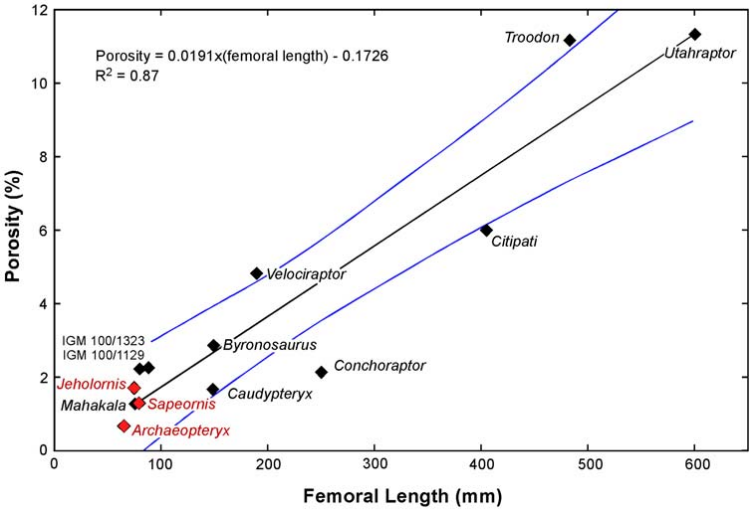
Maniraptoran femoral porosity shown with respect to scale. Intracortical transverse plane porosity from the histological sections was quantified (See Materials and Methods). These data are plotted with respect to femoral length. Data for non-avialan taxa are denoted by black diamonds. The regression equation describes only their distribution. The blue lines bound the 95% confidence interval. The red diamonds represent the basalmost avialans studied here. Note: their data are encompassed by the maniraptoran confidence interval and that for sister taxon Deinonychosauria (not shown).

Our analysis reveals that femoral histological patterning is correlated with size, and forms a scale-dependent continuum within Maniraptora (Figure 5). Moving from larger to smaller forms among the non-avialan dinosaurs, vascular complexity simplifies (plexiform to circumferential to longitudinal/reticular to longitudinal) and randomness in bone fiber orientation diminishes (woven to parallel-fibered). Deep cortical growth lines are variably present, but up to five exist in larger forms and no more than two in the smallest taxa. Femoral porosity decreases (11.32 to 1.26%) (Figure 6). Most notable from our analysis is that the smallest deinonychosaur taxon we sampled, *Mahakala*, (76 mm femoral length, [22]) lacks fibro-lamellar bone, but rather has reptilian-like parallel-fibered bone and shows sparse longitudinal vascularization (Figure 5).

**Figure 7:**
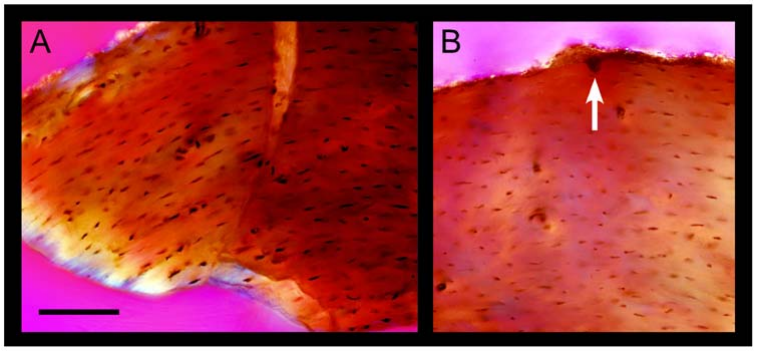
Histological section of an *Archaeopteryx* femur (BSPG 1999 I 50) viewed with polarized microscopy. (A) Parallel-fibered bone is found throughout the cortex as shown by the flattened, circumferentially oriented, lenticular osteocyte lacunae (tiny black structures) and matted bone fabric (lower left). (B) Primary longitudinal vascular canals are few (large black circular structures). These are occasionally found incompletely formed at the periosteal surface (arrow) and are responsible for the fibrous surface texture of the elements and long striae seen deep within the bones of all known individuals. Scale bar  = 0.75 mm.

Like *Mahakala*, histological analysis revealed that the femoral diaphysis of *Archaeopteryx* (BSPG 1999 I 50) is almost entirely composed of parallel-fibered bone with longitudinally oriented vascular canals (Figure 7). The latter are even more sparsely distributed (porosity 0.68%). Deep intracortical growth lines (lines of arrested growth and/or annuli) are absent in this young specimen [33]. A thin layer of endosteal bone is locally present. Haversian bone tissue was not found. The fibular metaphysis showed comparable primary bone histology, and compacted endosteal bone. The longitudinal vascular canals pervade throughout the semi-translucent cortices of the long bones. Partially formed canals manifest themselves as the conspicuous grooves seen at the periosteal surfaces (Figure 7).

**Figure 8:**
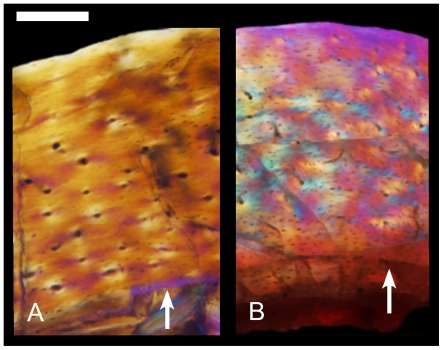
Femoral histology of the basal birds *Jeholornis* and *Sapeornis* viewed with polarized microscopy. (A) In *Jeholornis* (IVPP 13353), parallel-fibered bone matrix similar to that of *Archaeopteryx* makes up the cortex. However porosity is greater as in *Mahakala*. A growth line that locally varies between a line of arrested growth and an annulus is shown (arrow). (B) In the larger *Sapeornis* (LPM B00166), the matrix is primarily woven-fibered and shows a mix of longitudinal and reticular vascularization. Avascular parallel-fibered bone brackets a line of arrested growth (arrow) in this sub-adult specimen. Scale bar  = 0.15 mm

*Jeholornis* shows parallel-fibered bone and longitudinal vascularization like *Archaeopteryx* and the small deinonychosaur *Mahakala* (Figure 8). Woven bone is only locally present where the cortex is thickest. Femoral porosity is greater (1.7%) in this slightly larger taxon than *Archaeopteryx*. A single deep intracortical growth line is present in the largest individual (Figure 8). The even larger avialan *Sapeornis* shows primarily woven bone matrix and a mix of longitudinal and reticular vascularization similar to small oviraptorosaurs (e.g., *Caudipteryx*) and similar-sized deinonychosaurs (e.g., IGM 100/1323). Femoral porosity (1.3%) is also greater than that of *Archaeopteryx*. A single deep cortical growth line bounded by parallel-fibered bone separates growth zones in this sub-adult specimen (Figure 8).

The histological patterning (Figure 5) and porosity (Figure 6) of the basalmost birds *Archaeopteryx*, *Jeholornis*, and *Sapeornis* conforms to expectations based on the maniraptoran and deinonychosaur size continua. This supports the presence of a more complex size-dependent non-avialan histology but one that is nonetheless primitive for Avialae. Our results demonstrate that by the paravian diversification, when *Archaeopteryx*-sized dinosaurs evolved, parallel-fiber bone and reduced porosity was expressed.

**Figure 9:**
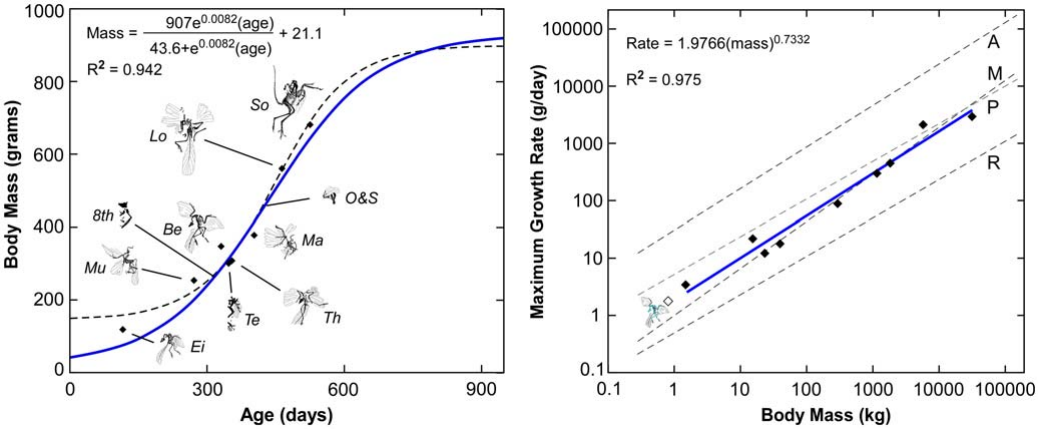
Growth depictions for *Archaeopteryx*. (Left) The size and estimated age for all ten specimens are depicted. The growth curves are based upon age and size estimates (diamonds) for the eight specimens where femoral length is known. The dashed line represents the best fit for the unconstrained statistical analysis with hatchling and adult size undefined. The solid line represents the best fit when hatchling and adult size are constrained. (Right) The maximal growth rates from these analyses (1.87–2.2 g/day; hollow diamond) fit expectations (1.83–1.87 g/day; [31]) for same-sized non-avialan dinosaurs (solid line) – animals that grew like slow growing endotherms, here compared to marsupials (M). The *Archaeopteryx* estimates are three times lower than typical rates for extant precocial land birds (5.7 g/day; P], 15 times lower than alticial land birds (28.6 g/day; A), and four times higher than typical rates for extant reptiles (0.46 g/day; R) [32]. Specimens designations: Ei  =  Eichstäat, Mu  =  Munich, 8^th^  =  8^th^ Exemplar, Te  =  Teyler, Th  =  Thermopolis, Be  =  Berlin, Ma  =  Maxberg, O&S  =  Exemplar der Familien Ottmann & Steil, Lo  =  London, So  =  Solnhofen.

Our growth analysis (see Materials and Methods) suggests that the known *Archaeopteryx* specimens span approximately 428 days in development (Figure 9). Maximal growth rates were in the range of 1.87 to 2.2 g/day across that span. These rates compare favorably with the expectations of 1.83 to 1.87 g/day for a same-sized non-avialan dinosaur (Figure 9) [31]. Asymptotic body mass was between approximately 895 and 928 g and somatic maturity appears to have been reached during the second to third years of life (Figure 9). The same timing is predicted for *Jeholornis* and *Sapeornis* based upon growth zone counts. These values are also consistent with expectations for outgroup non-avialan dinosaurs of comparable size [6].

## Discussion

Our discovery that the long bones of *Archaeopteryx* are composed of slow growing, reptilian grade, parallel-fibered bone is surprising. Well-vascularized woven bone was expected. The finding of the same peculiar matrix and vascular pattern in the slightly more derived bird *Jeholornis* indicates that the *Archaeopteryx* histology was not aberrant, but is typical of the basal avialan condition. The moderate increase in porosity in the birds *Jeholornis* and *Sapeornis* is concurrent with increased size, as is the predominance of woven matrix in the latter taxon.

Collectively the histological suites seen in the basalmost birds match expectations for same-sized taxa in the maniraptoran histological continuum. They provide compelling evidence that the non-avialan dinosaur growth pattern was inherited in totality and is typical of basal birds nearest to the *Archaeopteryx* node. This adds a physiological attribute to the long list of anatomical and behavioral attributes showing that birds are dinosaurs and that many early birds were typically very dinosaur-like [2]–[4]. Furthermore, it underscores that it is imperative to accommodate scaling when making phylogenetically grounded inferences about evolutionary changes in growth physiology through osseous histology [7].

The absence of available small outgroup taxa prior to this analysis likely hindered the detection of the precise relationship between body size and histology among paravians. Optimizing histological type within a broad sampling of maniraptoran theropods encompassing a diversity of sizes reveals multiple occurrences of the parallel-fiber suite within Paraves (Figure S2). Parallel-fiber bone growth, therefore, is not unique to Avialae. This is independent evidence that early avialan growth was neither novel, nor aberrant, but rather one end on the primitive histological continuum detailed above.

The ramifications of these findings for early avialan life history and physiology are considerable. For instance it has been recently speculated based on tissue characteristics (e.g. growth lines [5], [20], vascularization and fiber patterning [5], [9], [20]), broad size distribution (in *Archaeopteryx*; [2], [34]), and apparent age cohorts (in *Confuciusornis*; [35]) that growth in the first birds was slower than most same-sized living birds (Neornithes) which mature in just days to weeks [36]. However the estimates for the fossil birds range broadly from as little as two months to unspecified numbers of years.

We find more constrained estimates, whereby somatic maturity in the three basalmost lineages of birds occurred during the second and third years of life. Evidence for this includes: 1) the finding of exceptionally slow growing bone tissues suggesting prolonged development relative to living birds, 2) estimates that *Archaeopteryx* somatically matured in no less than 970 days (Figure 9) (there were 375 days in a Late Jurassic year; [37]), 3) growth lines that not only bridge the paravian record, but attest to developmental stoppages and multi-year development among the basalmost birds, and 4) histological character suites that meet expectations for same-sized non-avialan theropods known to mature in no less than two years [6].

Aside from anatomical differences such as long bony tails, clawed hands, and teeth, the slow development of the first birds would have made their biology appear unfamiliar to ornithologists. In most comparable-sized volant birds, juveniles and sub-adult sized animals are present for only a small fraction of the year as growth rates are remarkably rapid [1], [36]. In basalmost birds, actively growing juveniles would change imperceptibly week to week, and sub-adults would be present year-round. This slow development might also have prolonged their time to fledging (However what the volant capability was of early birds like *Archaeopteryx* remains conjectural [13], [16], [38]–[41].) Among same-sized living precocial birds individuals are typically earthbound for 3–6 weeks before becoming volant. Assuming that the age of the smallest *Archaeopteryx* specimens approximates when these animals first took flight across the Solnhofen lagoons, we can deduce that this milestone would have occurred no later than about 18 weeks (Figure 9).

Accurately estimating the adult size of basal birds is critical for studies of taxonomy, development, comparative physiology, ecology, heterochrony, flight biomechanics, and collectively the evolution of avialan success. This measure is known for *Jeholornis* and *Sapeornis* from specimens showing extensive neurocentral and braincase fusion. However for *Archaeopteryx*, our best representative of the initial avialan condition, adult size has remained ambiguous [33]. These animals have been variably described in the professional and lay public literature as robin, grackle, pigeon, magpie, small gull, crow, and chicken-sized. Quantified estimates of body mass have varied between 200 g and 600 g [39], [40]. Nevertheless, allometric scaling and skeletal fusion analyses have inferred that none of the known specimens are somatically mature and allude to a quantitatively larger endpoint [33]. Our histological and textural examinations showing that immature bone is still prevalent in the largest specimens strongly supports this (Figure 3), as do inferences from the growth curve that none are asymptotic individuals (Figure 9). The deinonychosaur histological continuum provides a new means to estimate the taxon's upper bound. It is unlikely that *Archaeopteryx* had a femoral length exceeding 75 mm as a shift to a more porous histology and perhaps woven matrix would have been evident (Figure 5). Femoral lengths of 70–75 mm translate to adult mass estimates of 822–1009 g, which can be analogized to the common raven (*Corvus corax*) for size [42].

Physiologically modern birds are endothermic and characteristically have among the highest relative growth rates and basal metabolic rates among extant animals [32]. Maximal growth rates strongly correlate with basal metabolic rate [32], [43]. Erickson and colleagues [31], [44], [45] have shown that whole body maximal growth rates for non-avialan dinosaurs are within the range expected for endotherms. These rates however are not typical of living birds. Rather they are nearer the lower bound for endotherms, and are more in line with animals such as marsupials [32], [43]. (Notably, kiwi (*Apteryx*), the living birds that show relative growth rates [46] most closely approaching the non-avialan dinosaurian condition, also have exceptionally low metabolic rates [47].) Since basal avialan body size, bone histology, and growth rates (as evidenced by *Archaeopteryx* and assumed for *Jeholornis* and *Sapeornis* through continuum matching) were retained from dinosaurian ancestry we can infer that these animals had a similar metabolism. This suggests that the initial conquest of the air was achieved using lower metabolic rates than are characteristic of today's avian flyers. It appears that the closest non-avialan relatives of birds were physiologically preadapted for powered flight and only anatomical adaptations were involved when birds first ventured into the air.

The stereotypical ideal of *Archaeopteryx* as a physiologically modern bird with a long tail and teeth has come under scrutiny in the last decade [2]–[4]. The present findings, based on the first direct histological evidence for *Archaeopteryx* and other basalmost birds, shows incontrovertibly that these animals were very primitive, similar to their non-avialan dinosaur precursors – *Archaeopteryx* was simply a feathered and presumably volant dinosaur. Theories regarding the subsequent steps that lead to the modern avian condition need to be reevaluated in a scale dependent manner to help understand what is turning out to be a complex evolutionary story.

## Materials and Methods

The taxonomic history of *Archaeopteryx* is complex, with almost every specimen having been assigned to a different species or even genus at some time (see [2] and [16], for recent reviews). However, Houck et al. [33] and Senter and Robins [48] argued that differences in proportions in nearly all specimens are explained by allometric developmental scaling. Other differences might be explained as sexual, or individual variation [2]. Thus, pending a more detailed revision of the taxonomy of *Archaeopteryx*, we agree with Chiappe [2] that all specimens are referable to a single species, *Archaeopteryx lithographica*.

The Munich specimen of *Archaeopteryx* (BSPG 1999 I 50) is preserved as slab and counterslab (Figure 4). From the fracture faces of the long bones we extracted minute cortical chips spanning the periosteal to endosteal surfaces (Figure 4; Figure S3). These included diaphyseal samples from the femur (the standard for comparative growth analyses in dinosaurs [5], [6] and a metaphyseal/diaphyseal sample of the fibula (an element that commonly preserves the majority of the growth record in theropods [6], [49]). Transverse plane histological slides were made and viewed with polarizing and reflectance microscopy and the microstructure described [50]. The periosteal fabric (seen in actual specimens and in research casts and photos, Table 1) and vascular patterning visible throughout the semi-translucent bones and on long bone fracture faces show that the juvenile histology of the Munich specimen is found throughout the growth series (Figure 3). This is not unexpected because all known *Archaeopteryx* are young individuals spanning a developmentally very limited range [33].

Diaphyseal histological sections and measurements of femora were then made for: the basal avialans *Jeholornis prima* and *Sapeornis chaochengensis*; the oviraptorids *Citipati osmolskae, Conchoraptor gracilis*, and *Caudipteryx zoui*; and the deinonychosaurs: *Mahakala omnogovae, Utahraptor ostrommaysi, Deinonychus antirrhopus, Velociraptor mongoliensis, Troodon formosus, Byronosaurus jaffei*, and two undescribed Mongolian troodontids (Table 1). The histology for deep cortical primary bone formed during early development was described. Intracortical transverse plane porosity was quantified using NIH Image J (Version 1.41o; National Institutes of Health, Bethesda, Maryland). Character mapping and phylogenetic optimization of these data was conducted using TNT version 1.0 [51] on a phylogeny adapted from Turner et al. [22] for Coelurosauria. From this we: 1) inferred the effects of scaling on histological patterning with femoral length as a size standard, 2) predicted the primitive character state for Paraves, and 3) inferred whether evolutionary changes occurred across the transition.

Because the *Archaeopteryx* specimens are young individuals that fortuitously span middle development [33] it was possible to estimate age differences between individuals and speculate on *Archaeopteryx* growth rate during the exponential stage of development. This was achieved using neontologically derived tissue formation rates, similar to the means described by Sander and Tückmantel [30] for aging sauropods. Differences in antero-posterior femoral midshaft diameter relative to the Munich specimen were determined throughout the growth series. (In specimens with uncrushed femora the ratio of femur length to diameter ranges from 13.47–13.67 across ontogeny (Eichstätt-London specimens). The mean ratio of 13.62 was used to deduce the diameter for the Solnhofen specimen (BMMS 500).) Radial tissue formation rates derived from femora in living birds [52], [53] were divided into these measurements and the age differences deduced. Positive correlations between tissue formation rates and body size among homologous elements are evident in birds [52], [53]. Sustained radial formative rates for the matching histological type to *Archaeopteryx* avian femora is 2.5 um/day in *Archaeopteryx*-sized birds (e.g. 880 g Mallard ducks; *Anas platyrhynchos*). Rates as high as 4.2 um/day occur in very large taxa (e.g. 90 kg Ostrich, *Struthio camelus*) for femora showing the same vascularizaton pattern, however the matrix is woven-fibered. The former value was used.

Body mass of the large Solnhofen specimen (BMMS 500) was determined using the femoral and tibial regression equations for galliforms (“poor flying birds”) from Alexander [54]. The Developmental Mass Extrapolation scaling principle [6], [44] was used to infer specimen masses and coupled with the age differences to model growth rates throughout development. We first used maximum likelihood to fit a logistic equation with normal error distribution to the growth data for the eight *Archaeopteryx* specimens for which femoral length is known (mass  =  (a*exp[b*age])/(*c*+exp[b*age]) + *d*, *a* = 752.6, *b* = 0.0118, *c* = 274.2, *d* = 145.7, r^2^ = 0.964). This growth curve projects an estimated hatchling mass of 148 g, an adult mass of 898 g, and a maximal daily growth rate of 2.2 g/day. Because the projected hatchling mass from this maximum likelihood estimate is biologically implausible, we used comparisons with other taxa and our histological observations to construct informative prior distributions for hatchling and adult mass and calculated a Bayesian estimate of *Archaeopteryx* growth. Specifically, a 39.6 g estimate for a full-term egg mass was obtained from the regression analysis of Blueweiss [55]. A similar size of 53 g was predicted using the femoral dimensions for a near term *Gobipteryx minuta* (IGM 100/1291) scaled up to account for the larger size of *Archaeopteryx*. The femoral histological continuum [see results] predicts that *Archaeopteryx* had a femoral length that was less than 75 mm otherwise the histology seen in larger paravians would have been expressed. The largest known *Archaeopteryx* is 683 g and the slightly larger paravians, which have a modified histology owing to scale dependence, place full adult size at less than 1009 g. Based on these estimates we used informative priors for hatchling mass of approximately 43±10 g (*d* = Normal[20, sd = 5]) and an adult mass of 895±165 g (*a* = Normal[875, sd = 82.5]). This prior distribution for adult mass allows for a large range of plausible adult sizes, whereas the estimated hatchling size is constrained to a smaller range. Maximum daily growth rate was unconstrained, as we used uninformative priors for parameters *b* and *c*(Gamma [0.01, 0.01]). The Bayesian analysis provided similar results to the unconstrained maximum likelihood fit, but more plausible posterior estimates of hatchling size (41.4 g) and adult size (928 g) and a slightly lower estimate of maximal growth rate at 1.87 g/day (*a* = 907±71.5, *b* = 0.0082±0.0008, *c* = 43.6±25, *d* = 21.1±5, means±sd, r^2^ = 0.942). The results of these growth analyses were compared with predictions for same-sized non-avialan dinosaurs and extant precocial birds.

Statistical analyses were conducted using R 2.9.0 (R Development Core Team; R Foundation for Statistical Computing, Vienna, Austria) and WinBUGS 1.4.3 [56].

Although it is plausible that hatchling *Archaeopteryx* initially grew using woven bone as has been shown in enantiornithines [11], [20], this would have had negligible affect on the maximal growth rate estimates made here since the studied specimens span the entire exponential stage of development (Figure 9). Furthermore, very little development occurred prior to the size obtained by the Eichstätt specimen (JM 2257), just 78 grams. So individual longevity estimates would be diminished by no more than approximately 24–30 days from initial woven bone formation. Conversely, growth stoppages in the largest *Archaeopteryx* specimens would have contributed to moderately higher longevity estimates for these specimens and slightly lower maximal growth rate estimates. This would bolster the overall conclusion that basal avialans were not growing like living precocial birds (see results).

## Supporting Information

Figure S1 Preferred topology for Maniraptora used in the present study. Tree topology is derived from the phylogenetic analyses of Turner et al. [22]. Taxa listed in bold are those for which we obtained histological data.
(1.19 MB TIF)

Figure S2 Optimization of fiber types on femoral length. Optimization of bone fiber-type on maniraptoran phylogeny. Woven bone fiber-type is widespread among maniraptorans. Note that minimally two, but perhaps three, independent acquisitions of parallel fiber bone occurred in the small paravians Mahakala, Archaeopteryx, and Jeholornis.
(1.14 MB TIF)

Figure S3 Long bone chips extracted from the Munich Archaeopteryx (BSP 1999 I 50).
(2.52 MB TIF)
