# Supplementary figures and images for: Was Dinosaurian Physiology Inherited by Birds? Reconciling Slow Growth in Archaeopteryx
Source: PLoS One. 2009 Oct 9;4(10):e7390. doi: 10.1371/journal.pone.0007390 (PMC2756958; doi:10.1371/journal.pone.0007390)

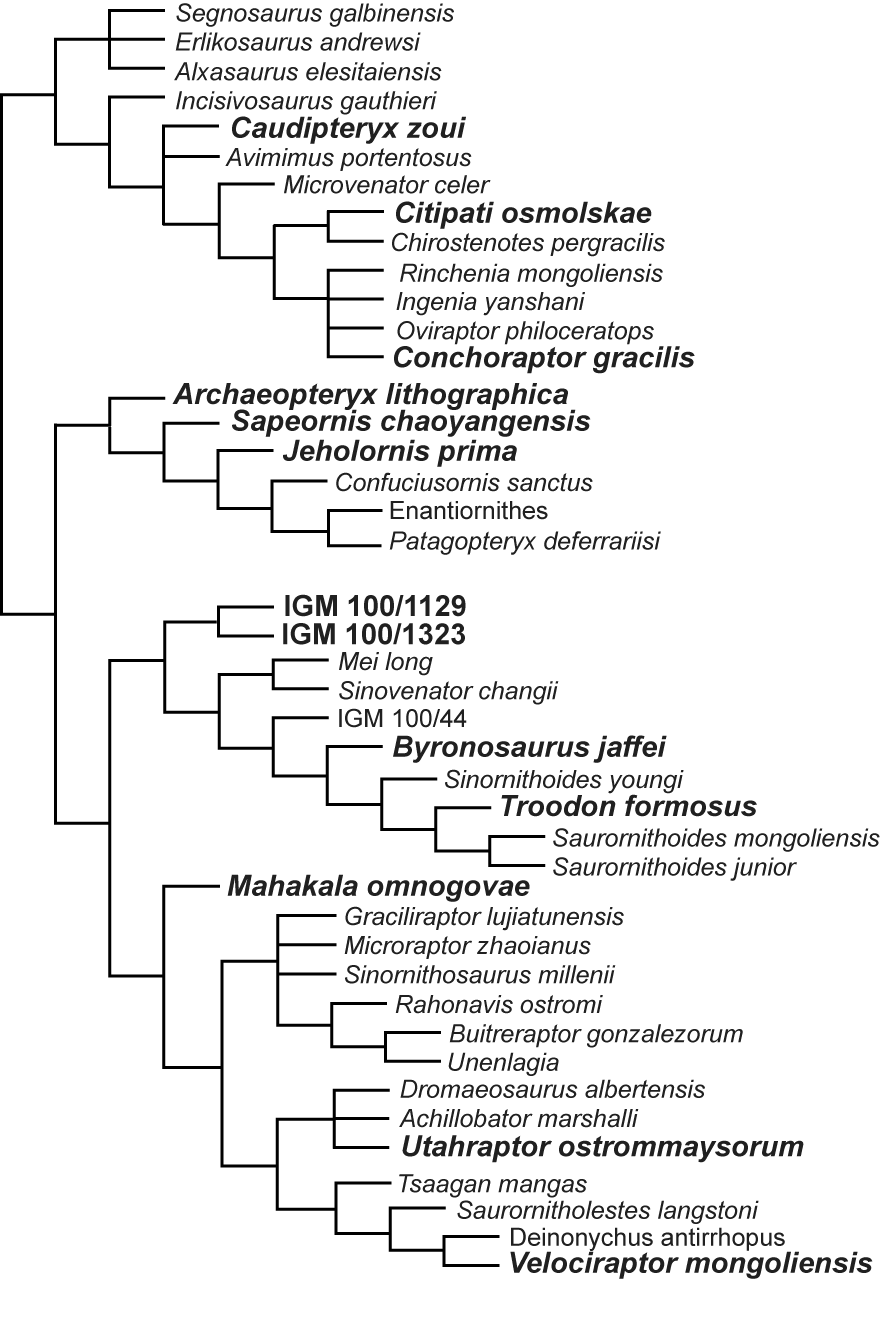

Supplement: Figure S1 — Preferred topology for Maniraptora used in the present study. Tree topology is derived from the phylogenetic analyses of Turner et al. [22]. Taxa listed in bold are those for which we obtained histological data. (1.19 MB TIF) [file pone.0007390.s001.tif]

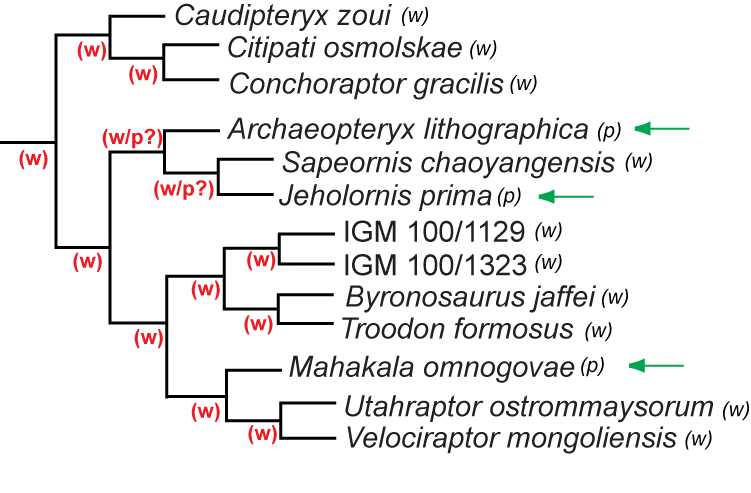

Supplement: Figure S2 — Optimization of fiber types on femoral length. Optimization of bone fiber-type on maniraptoran phylogeny. Woven bone fiber-type is widespread among maniraptorans. Note that minimally two, but perhaps three, independent acquisitions of parallel fiber bone occurred in the small paravians Mahakala, Archaeopteryx, and Jeholornis. (1.14 MB TIF) [file pone.0007390.s002.tif]

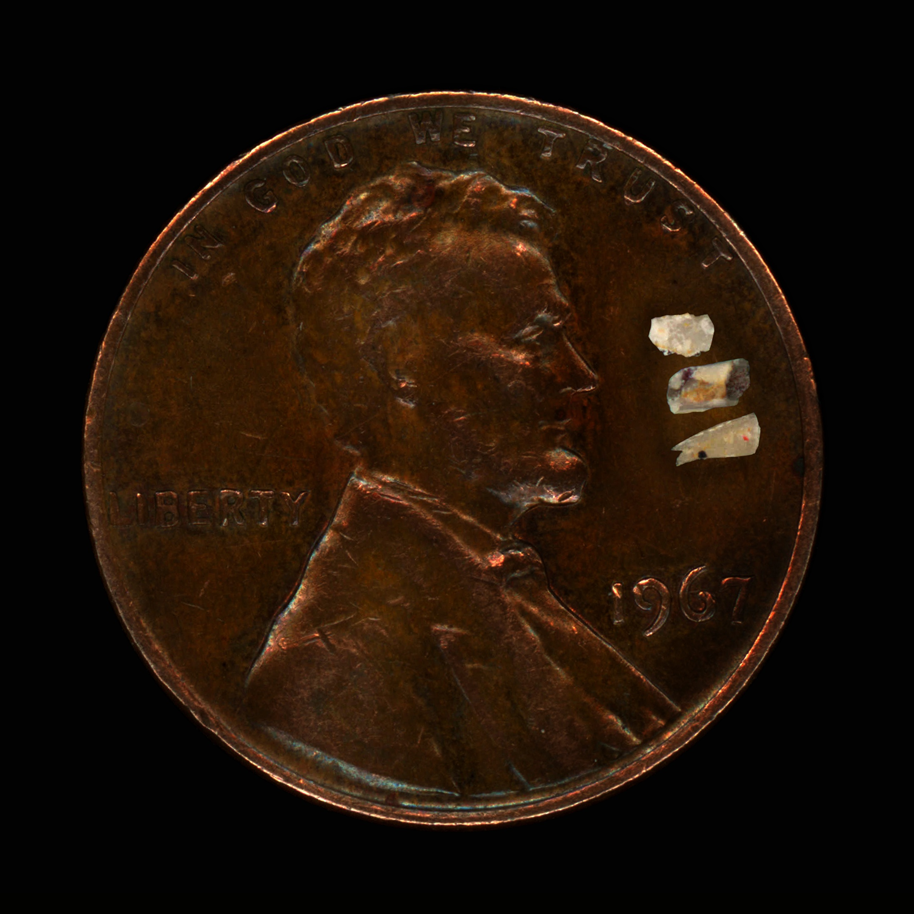

Supplement: Figure S3 — Long bone chips extracted from the Munich Archaeopteryx (BSP 1999 I 50). (2.52 MB TIF) [file pone.0007390.s003.tif]
